# Characteristics and outcomes of older people on antiretroviral therapy in Tlokwe Clinics, South Africa

**DOI:** 10.4102/sajhivmed.v21i1.1066

**Published:** 2020-07-07

**Authors:** Mareike Rabe, Huibrecht C. Lion-Cachet, Melaku A. Eyassu

**Affiliations:** 1 Department of Family Medicine and Primary Care, Faculty of Health Sciences, University of the Witwatersrand, Johannesburg, South Africa

**Keywords:** HIV, older adults, characteristics, outcomes, antiretroviral therapy

## Abstract

**Background:**

South Africa (SA) has a large human immunodeficiency virus (HIV) epidemic but little is known of its effect on those ≥ 60 years of age viz. ‘older-persons’ living with HIV (OPLWH). Numbers in this age group are increasing and are expected to place a greater strain on existing resources.

**Objectives:**

To describe the demographic features and the co-morbidities of OPLWH in Tlokwe. This included an assessment of viral load (VL) suppression and the identification of associations between patient characteristics and clinical outcomes.

**Methods:**

A retrospective file review was undertaken to cover the period 01 May 2017 to 30 April 2018. Descriptive statistics were applied to demographic and clinical data and to treatment outcomes. Statistically significant associations were subjected to logistic regression analysis.

**Results:**

Of the 191 participants, 111/191 (58.1%) were female and 167/191 (87.4%) were 60 –70 years of age. Of the participants, 154/191 (81.9%) were virally suppressed (< 400 copies/mL). Hypertension (*n* = 106/191, 55.5%) was the most frequently identified co-morbidity. A CD4 cell count of ≥ 350 cells/mm^3^ at last assessment correlated positively with VL suppression (odds ratio 2.3, confidence interval 1.05–5.02, *p* = 0.037).

**Conclusion:**

Although the level of VL suppression in this cohort was high, greater effort is required to bring this in line with the Joint United Nations Programme on HIV/AIDS (UNAIDS) recommendations viz. 90% viral suppression in PLWH by 2030. Further research is needed to define the evolving long-term needs of OPLWH and to facilitate entry into care of those currently not in care.

## Introduction

The term ‘older-persons’ is defined by the United Nations (UN) as persons aged ≥ 60–65 years.^1^ This definition has been used in this study because South African citizens become eligible for an ‘older-persons’ government grant at 60 years of age.^2^ Globally, the number of people in this age group is increasing. Over the next 30 years, a growth rate of 218% of older persons, that is from 32 million in 2019 to 101 million in 2050, is predicted for sub-Saharan Africa (SSA).^1^ Over the same period, South Africa’s (SA) older-person population is likely to increase from 8% currently, to 10.5% in 2030, and to 15.4% in 2050.^3^ The drivers of this change include lower fertility rates (improved access to contraception), reduced childhood mortality and better access to healthcare for all.^4^ The number of people living with human immunodeficiency virus (HIV) (PLWH) in SA – including older persons living with HIV (OPLWH) – will also increase. These survival gains have followed the widespread use of antiretroviral therapy (ART) and the implementation of government programmes such as Universal Test and Treat (UTT).^5^ For many, HIV infection has become a chronic condition.^6^

South African doctors are ill equipped to provide care to OPLWH, as they are not adequately trained in gerontology^7^ and trained geriatricians in SA tend to work in academic centres. Medical training centres in SA do not offer sub-speciality training that combines geriatrics and HIV. The lack of trained infectious disease specialists in the sphere of geriatric medicine may pose a problem in future, and primary care clinicians will likely treat these patients, as they do currently. The treatment and care OPLWH need is laborious and complex. The persistence of subclinical inflammation even when a patient is on effective ART leads to persistent immune dysfunction. This is believed to result in cardiovascular dysfunction, end-organ failure and non-AIDS defining malignancy viz. the increase of co-morbid conditions. Old age increases health risks. These include multiple pathologies: drug-drug interactions and the predisposition to drug-toxicity (renal and liver), greater likelihood of neurovascular impairment, frailty and fractures, limited immune recovery (especially CD4 cell count), less social security (finances and support) and greater dependency on the state and local health services.^8^ On the other hand, OPLWH are usually adherent to treatment and likely to follow up at their health care centres. They are likely to remain in their current residence and, thus, are generally contactable. In light of the above, it is necessary for researchers in SA to understand this cohort in all settings in order to develop feasible and sustainable care packages. There is a paucity of data for OPLWH in the SA context.

Identifying HIV in the elderly is important. Benefits include reducing the risk of transmission as many are still sexually active and practise poor prevention strategies. Early entry into care will reduce the risk of opportunistic conditions and aid in the timely diagnosis of co-morbidities. In the SA public sector, OPLWH have access to specialised healthcare and ART at minimal cost. If retained in-care, costs such as hospitalisation and end-of-life care are more readily anticipated and mitigated.^8^ Nonetheless, immune reconstitution is often incomplete in this group, despite ART and viral suppression, particularly where presenting nadir CD4 levels, were low.^8^

Two regional studies provide data on the prevalence and outcome of HIV infection in SA-OPLWH.^9,10^ A retrospective study of 7295 OPLWH (study-total *n* = 83 566 PLWH) who started ART between 2004 and 2013 found that those ≥ 50 years of age increased from 6% in 2004 to 10% in 2012/2013. These finally constituted 9% of the entire cohort.^9^ This study assessed only three of SA’s nine provinces and, in particular, excluded the North West Province (NWP). A second study confirmed a significantly higher prevalence of diabetes mellitus (DM) and hypertension (HPT) in its OPLWH: DM, *n* = 16/262 (6.3%) versus 24/3741 (0.7%) and HPT, *n* = 55/262 (21.5%) versus 79/3741 (2.2%), *p* < 0.001, respectively.^10^

The prevalence of HIV infection in Tlokwe’s older citizens is unknown. Those aged ≥ 65 years comprise 5.7% of the population of the town.^11^ Data from the 2019 mid-year population census described only the 15–49 year age group.^12^ In 2012, the Human Sciences Research Council (HSRC) reported the general prevalence of HIV in South Africans of ≥ 50 years to be 7.6% (95% CI 6.5–8.8).^13^

 The objectives of this study were the following:

- To describe the demographic characteristics of the study population
- To describe their co-morbidities
- To assess viral load (VL) suppression rates
- To determine the relationship, if any, of patient characteristics to the following four outcomes viz.: VL suppression and immune (CD4 cell count) recovery on ART, loss to follow-up (LTFU) and death.

## Research methods and design

### Study design and study population

This was a retrospective study of OPLWH undertaken to cover the period 01 May 2017 to 30 April 2018 at three healthcare sites in Tlokwe (Potchefstroom), a town in the NWP of SA. The study subjects were aged ≥ 60 years and were on ART for a minimum of one year prior to the period under review, hence, they had to be initiated on ART prior to 30 April 2017. The three clinics were chosen to represent, as closely as possible, the diversity of people who utilise the public health service in the town.

**Clinic A:** a primary care clinic situated close to the municipal hospital that serves residents and patients referred from hospital clinics. Services provided by the clinic include counselling, HIV testing and the initiation of treatment of HIV. Afrikaans and Setswana are spoken by the majority of patients accessing clinic services.

**Clinic B:** a community health centre serving a predominantly Setswana-speaking population.

**Clinic C:** a community health centre serving both Afrikaans and Setswana-speaking residents.

### Inclusion criteria

- Older persons living with HIV aged ≥ 60 years at their last birthday and with the time of their latest clinic visit being between 01 May 2017 and 30 April 2018.
- Older persons living with HIV who had been on ART for at least 12 months prior to the study census that is to include as a minimum a second VL test whilst on ART.^14^
- Confirmation as an adherence measure, that the patient or a nominated representative of the patient, had collected treatment on their behalf, at the regular three-monthly medicines collection visits in the year under study.

### Exclusion criteria

- Patient clinic file ‘missing’ for the study period under review.
- Patients who did not meet the inclusion criteria.

## Data collection

A collection sheet developed by the researcher was used to capture data as per the study’s objectives. All information was captured manually and patient identifiers were removed. Data were then transferred to Excel 2016 (Microsoft, US). Study subjects comprised all available patients recruited from the study sites who fulfilled entry criteria. A prior calculation of the ‘sample size’ was not undertaken. All files were retrieved with the help of data capturers and clerks at the research sites. TIER.Net.,^15^ an electronic patient management system, was used to access additional (missing) patient information. Further laboratory data were located on the TrakCare service of the SA National Health Laboratory Services (NHLS). Some data that were more than 1 year old, were excluded as these did not represent the patient’s status at the time of the study census. These data included laboratory values of creatinine, haemoglobin and total cholesterol blood results.

## Data analysis

A list of study definitions can be found in Appendix 1. Stata/IC 16.0 software (STATA Corporation, LLC, TX, US 2019) was used to analyse data. For descriptive statistics numbers, percentages, medians, minimum and maximum values and interquartile ranges (IQR) were used. For evaluating associations between demographic, clinical and laboratory characteristics with four treatment outcomes viz. the most recent VL and CD4 cell count, LTFU and death, Pearson’s chi-square, Fisher’s exact, Mann–Whitney (Wilcoxon rank-sum), Kruskall-Wallis or Spearman’s correlation tests were used where appropriate. Most values for the continuous variables were shown to be non-normally distributed as per the Shapiro–Wilk normality test. Even after logarithmic transformation, the data were non-normally distributed. All variables were initially assessed individually against the treatment outcomes. If *p* values were ≤ 0.05 or there were > 0.5 correlations for measures of association, these predictors were entered into a linear or logistic regression model where appropriate. A level of ≤ 0.05 was considered statistically significant. During the initial analysis, the VL and latest CD4 cell count outcomes were assessed as continuous variables as well as clinical categories. Whenever there was a statistically significant relationship between any continuous or categorical outcome in the initial analysis, it was used in the binomial logistic regression model provided that the number of observations was ≥ 10 per cell. The binomial logistic regression model categorised the VL as suppressed (< 400 copies/mL) or unsuppressed (≥ 400 copies/mL) and the latest CD4 cell as high (≥ 350 cells/mm^3^) or low (< 350 cells/mm³).

### Ethical consideration

Ethical clearance was granted by both the Human Research Ethics Committee (Medical) of the University of the Witwatersrand, Johannesburg (M180304) and the North West Health Research Committee (NW_2018_006). The Tlokwe sub-district Office of the Primary Health Care Manager also granted permission for the research. Owing to the retrospective nature of the study, patient’s anonymised data were evaluated. Consequently, informed consent was waived for this study.

## Results

Data from a total of 191 clinic files of OPLWH were examined. Clinic A provided *n* = 38/191 (19.9%), Clinic B, *n* = 96/191 (50.2%) and Clinic C, *n* = 57/191 (29.8%). One patient file from Clinic A had to be excluded as this was a duplicate file. Eleven patient files were missing from Clinic B, as were 12 files from Clinic C. An overview of the demographic, treatment, clinical and laboratory characteristics is provided in Table 1. All participants were pensioners.

**TABLE 1:** Demographic, treatment, clinical and laboratory characteristics.

Demographics	*n*	*N*	%	Minimum	Maximum	Median	IQR
**Gender**							
Female	111	191	58.1	-	-	-	-
Male	80	191	41.9	-	-	-	-
**Marital status**							
Married/Partner/Cohabitation	53	191	27.8	-	-	-	-
Single/Widowed	39	191	20.4	-	-	-	-
Unknown	99	191	51.8	-	-	-	-
**Previously defaulted treatment**	18	191	9.4	-	-	-	-
Male	9	18	50	-	-	-	-
Single/Widowed	5	18	27.8	-	-	-	-
Still receiving first-line ART	18	18	100	-	-	-	-
VL < 400 copies/mL	13	18	72.2	-	-	-	-
**Transferred out**	8	191	4.2	-	-	-	-
**ART regimen**							
ART first line (usual): Tenofovir/emtricitabine/efavirenz	158	191	82.7	-	-	-	-
ART second line (usual): Zidovudine/lamivudine/lopinavir/ritonavir	10	191	5.2	-	-	-	-
ART first line (alternative): Abacavir or zidovudine/lamivudine/efavirenz	21	191	11	-	-	-	-
ART first line (alternative): Tenofovir/emtricitabine/nevirapine	2	191	1.1	-	-	-	-
**Hospitalised in the previous 12 months**	8	191	4.2	-	-	-	-
CD4 cell count < 200 cells/mm³	2	8	25	-	-	-	-
Current TB	1	8	12.5	-	-	-	-
Co-morbidity	7	8	87.5	-	-	-	-
**Co-morbidity noted†**	123	191	64.4	-	-	-	-
**Tuberculosis**							
Current	6	191	3.2	-	-	-	-
Previous	5	191	2.6	-	-	-	-
**Hypertension**	106	191	55.5	-	-	-	-
Creatinine ≥ 100 µmol/L	18	92	19.6	-	-	-	-
eGFR ≤ 50 mL /min/1.73m³	5	92	5.4	-	-	-	-
**Diabetes Mellitus**	15	191	7.9	-	-	-	-
Creatinine ≥ 100 µmol/L	1	14	7.1	-	-	-	-
eGFR ≤ 50 mL /min/1.73m³	0	14	0.0	-	-	-	-
**Chronic Kidney Disease**	7	191	3.7	-	-	-	-
Creatinine ≥ 100 µmol/L	5	7	71.4	-	-	-	-
eGFR ≤ 50 mL /min/1.73m³	4	7	57.1	-	-	-	-
**Hypercholesterolaemia**	18	191	9.4	-	-	-	-
**Age (years)**							
60–69	167	191	87.4	-	-	-	-
≥ 70	24	191	12.6	-	-	-	-
**Duration on ART (months)**							
12–60	76	191	39.8	-	-	-	-
61–120	66	191	34.6	-	-	-	-
≥ 121	39	191	20.4	-	-	-	-
Unknown	10	191	5.2	-	-	-	-
**Weight (kg)**							
< 40	5	191	2.6	-	-	-	-
40–59	68	191	35.6	-	-	-	-
60–79	78	191	40.8	-	-	-	-
≥ 80	33	191	17.3	-	-	-	-
Unknown	7	191	3.7	-	-	-	-
**VL (copies/ml)**				LTDL	394 143	20	0 – 157.5
< 400	154	191	80.6	-	-	-	-
400–999	16	191	8.4	-	-	-	-
≥ 1000	18	191	9.4	-	-	-	-
Unknown	3	191	1.6	-	-	-	-
**Initial CD4 cell count (cells/mm³)**				6	1118	279.5	167 – 433
< 200	67	191	35	-	-	-	-
200–349	54	191	28.3	-	-	-	-
350–499	26	191	13.6	-	-	-	-
≥ 500	37	191	19.4	-	-	-	-
Unknown	7	191	3.7	-	-	-	-
**Latest CD4 cell count (cells/mm³)**				51	1873	536	337.5 – 703.5
< 200	19	191	9.9	-	-	-	-
200–349	30	191	15.7	-	-	-	-
350–499	31	191	16.2	-	-	-	-
≥ 500	104	191	54.5	-	-	-	-
Unknown	7	191	3.7	-	-	-	-
**Creatinine (µmol/L)**				**29**	**632**	**81**	**65** –** 98**
≤ 99	121	191	63.4	-	-	-	-
≥ 100	36	191	18.8	-	-	-	-
Unknown	34	191	17.8	-	-	-	-
**eGFR (mL /min/1.73m³)**							
≤ 50	13	191	6.8	-	-	-	-
> 50	144	191	75.4	-	-	-	-
Unknown	34	191	17.8	-	-	-	-

IQR, interquartile range; WBOT, ward-based outreach team; ART, antiretroviral therapy; TB, tuberculosis; VL, viral load; LTDL, lower than detectable level. eGFR, estimated glomerular filtration rate as per the Chronic Kidney Disease Epidemiology Collaboration Equation (CKD-EPI).

† , Participants had up to three chronic conditions. Cardiovascular diseases (myocardial infarction and stroke) and malignancies were not studied.

## Antiretroviral therapy treatment, clinical and laboratory characteristics

Out of 157 participants for whom the ART regimens and the latest serum creatinine blood results were available, some 12/150 participants (8%) had an eGFR of ≤ 50 mL/min/1.73m³ whilst on first-line ART. Those on second-line ART showed a low eGFR in 1/7 participants (14.3%). Not a single participant with a serum creatinine of ≤ 100 µmol/L had renal dysfunction as per the chronic kidney disease (CKD)- Epidemiology Collaboration Equation (EPI) equation, whereas 13/36 (36.1%) of those with a creatinine of ≥ 100 µmol/L had renal dysfunction, according to this equation.

Twenty-nine out of 34 participants (85.3%) with an unsuppressed VL (≥ 400 copies/mL) remained on a non-nucleoside reverse transcriptase inhibitor (NNRTI)-based ART regimen, whereas 5/34 (14.7%) were on a second-line regimen with a protease inhibitor (PI) boosted with ritonavir. The PI was lopinavir in all cases. Of the participants with a VL of 400 copies/mL – 999 copies/mL, 14/16 (87.5%) were on a NNRTI first-line regimen, whereas for those with a VL of ≥ 1000 copies/mL, 15/18 (83.3%) were on a NNRTI first-line regimen.

The median CD4 cell count improved from a baseline of 279.5 cells/mm³ (IQR 167–433) to 536 cells/mm³ (IQR 337.5–703.5) at the most recent CD4 cell count monitoring visit, resulting in a median improvement of 256.5 cells/mm³. Moreover, some 33% of the cohort had a CD4 cell count of ≥ 350 cells/mm³ at baseline compared to 70.7% at the most recent CD4 cell count monitoring visit. Close to 10% of the cohort still had a CD4 cell count of < 200 cells/mm³ at the latest CD4 cell count monitoring visit. For this group, 10/19 (52.3%) participants had an unsuppressed VL of ≥ 400 cells/mL. This group also showed that 12/19 (63.2%) suffered from a defined co-morbidity. The majority of this group (*n* = 16/19, 84.2%) were on first-line ART.

Recent haemoglobin blood results were available for 24 participants. Of these, 4/24 (16.7%) had a haemoglobin of < 8g/dL, whilst 3/24 (12.5%) had a haemoglobin of 8 g/dL – 12 g/dL and 17/24 (70.8%) had a haemoglobin of ≥ 12g/dL. Total cholesterol blood results were available for 75 participants. Of these, 40/74 (53.3%) had readings of < 5 mmol/L and 35/74 (46.7%) had readings of ≥ 5 mmol/L. For participants who were classified with hypercholesterolaemia, 9/14 (64.3%) had recent total cholesterol readings of ≥ 5 mmol/L whereas 26/61 (42.6%) participants who were not classified to have hypercholesterolaemia had recent total cholesterol readings of ≥ 5 mmol/L.

## Co-morbidities

One or more co-morbidity was found in 123/199 (64.4%) participants. Eighty-four participants (44%) had one chronic condition, a number of 34 participants (17.8%) had two chronic conditions and five participants (2.6%) had three chronic conditions. The majority of the participants (*n* = 106/191, 55.5%) had HPT. The following other co-morbidities were found: hypercholesterolaemia (*n* = 18/191, 9.4%), DM (*n* = 15/191, 7.9%), CKD (*n* = 7/191, 3.7%), a mental health problem (*n* = 6/191, 3.1%), asthma (*n* = 5/191, 2.6%), post-herpetic neuralgia (PHN) (*n* = 2/191, 1.0%), epilepsy and chronic obstructive airway disease (*n* = 1/191, 0.5%). Six participants (3.1%) had tuberculosis (TB) during the study period and five participants (2.6%) previously had TB. Renal dysfunction was found in 4/7 (57.1%) participants who were classified with CKD.

## Virologic suppression

The VL results were available for all but three participants in the cohort (98.4%). Female participants showed 81.7% VL suppression rates compared to 82.3% for male participants. The overall VL suppression rate was 81.9%. Table 2 depicts the proportion of participants with VL suppression considering their most recent CD4 cell counts. Nineteen per cent of participants still had a CD4 cell count of < 350 cells/mm³, even when they were found to have VL suppression. One hundred and eleven participants (59%) had a VL of ≤ 50 copies/mL.

**TABLE 2:** Most recent viral load and CD4 cell counts.

Variable	CD4 < 350 cells/mm³		CD4 ≥ 350 cells/mm³		Total	
	*n*	%	*n*	%	*n*	%
VL < 400 copies/mL	35	19	115	62.5	150	81.5
VL ≥ 400 copies/mL	14	7.6	20	10.9	34	18.5

**Total *n* (%)**	**49**	**26.6**	**135**	**73.4**	**184**	**100**

VL, viral load.

## Relationships between patient characteristics and outcomes

There were no strong correlations or meaningful linear regression results amongst the most recent VL, latest CD4 cell count and other continuous variables (age, duration of ART in months, weight, baseline CD4 count, creatinine, haemoglobin and total cholesterol). During the study period, six participants were LTFU (3.1%) and four died (2.1%) with a mean age of 65.5 years at death. No statistically significant relationships were evident for the outcome LTFU. Table 3 shows the statistically significant relationships that were apparent between patient characteristics and outcomes. Hospitalisation in the past year was positively correlated with dying (50% of participants who died during the study period also had been hospitalised in that year). Moreover, participants with lower haemoglobin levels (< 8 g/dL) had a greater association with dying than those with higher haemoglobin levels where there were no mortalities. Statistically significant relationships for VL were: firstly, participants tended to have higher CD4 cell counts when their VL was suppressed and, secondly, PHN was associated with an unsuppressed VL, although only two participants in the cohort were noted to have PHN. Participants on first-line ART had 2.78 greater odds of having higher CD4 cell counts, whereas those on ART for > 5 years had 3.15 greater odds of having higher CD4 cell counts. Although not statistically significant in the logistic regression model, it is still worth noting that female participants had 2.24 times higher odds of having higher CD4 cell counts than male participants and that the odds of having a lower creatinine were 1.67 times more likely in those with higher CD4 cell counts. A high baseline CD4 cell count was associated with a high recent CD4 cell count, and current TB infection was associated with lower recent CD4 cell counts.

**TABLE 3a:** Relationships between patient characteristics and outcomes.

Outcome	Alive	Dead	Chi-square/Fisher exact	Logistic regression		
Characteristic	*N*	*n*	*p* value	*p*	OR	95% CI
Hospitalised in the past year	6	2	0.009	n/a†	-	-
Haemoglobin < 12 g/dL	3	4	0.017	n/a†	-	-

**TABLE 3b:** Relationships between patient characteristics and outcomes.

Outcome	VL ≤ 400 copies/mL	VL > 400 copies/ml	Chi-square/Fisher exact	Logistic regression		
Characteristic	*N*	*n*	*p* value	*p*	OR	95% CI
Current CD4 ≥ 350 cells/mm^3^	115	20	0.034	0.037	2.3	1.05–5.02
Post-herpetic neuralgia	0	2	0.032	n/a†	-	-

**TABLE 3c:** Relationships between patient characteristics and outcomes.

Outcome	CD4 < 350 cells/mm³	CD4 ≥ 350 cells/mm³	Chi-square/Fisher exact	Logistic regression		
Characteristic	*N*	*n*	*p*	*p*	OR	95% CI
Patient on first-line ART	35	116	0.023	0.041	2.78	1.04–7.42
Patient on ART > 5 years	19	83	0.007	0.009	3.15	1.34–7.40
Baseline CD4 ≥ 350 cells/mm^3^	8	54	0.002	n/a†	-	-
Female gender	19	89	0.001	0.059	2.24	0.97–5.18
Current tuberculosis	4	1	0.018	n/a†	-	-
Creatinine ≤ 99 µL/L	23	96	0.012	0.277	1.67	0.66–4.18

OR, odds ratio; CI, confidence interval; VL, viral load; ART, antiretroviral therapy.

† , Cells contain ≤ 10 observations, thus findings were omitted from the logistic regression model.

## Discussion

According to our knowledge, this study was the first to describe patient characteristics and outcomes of PLWH ≥ 60 years old on ART in Tlokwe. Participants 60–69 years old and female participants comprised the largest proportion of our sample. The VL suppression rate in this cohort needs to be improved. Almost two-thirds of our sample had one or more co-morbidity. As a small proportion of participants were LTFU or had died during the study period, it is unsurprising that there were few significant relationships between these outcomes and patient characteristics. In this cohort, a suppressed VL was associated with a good CD4 cell response. A good CD4 cell response was also associated with first-line ART and longer treatment duration.

Participants 60–69 years old and female participants comprised the largest proportion of this study’s sample. Female gender seemed to predominate in South African studies evaluating OPLWH from the age of 50 years old.^10,16,17^ Interestingly, the only other study in SSA that showed a female predominance was a recent Ugandan study.^18^ Studies from other countries in SSA painted a slightly different picture. Auld et al.^19^ pooled data from seven SSA countries. Male participants aged ≥ 50 years formed the majority of the cohort in six of the seven countries that were studied. Nigeria was the marginal exception, where 51% of participants ≥ 50 years old were female participants. A male predominance was also evident in Burkina Faso^20^ and Malawi.^21^ Both studies used the age of ≥ 50 years to define older adults.

In 2012, the SA National HIV prevalence incidence and behaviour survey reported a higher HIV prevalence in female participants aged 55–60 years, but male participants still predominated in the age categories of 50–55 years and ≥ 60 years, in contrast to our findings. In the aforementioned survey, the proportion of men who reported having more than one sexual partner was eight times that of women. Older men also reported low condom use during their last sexual encounter. These factors may explain why men are more likely to become HIV infected when they are older. On the other hand, women are known to test for HIV more readily than men and are more aware of their status, which could increase their HIV incidence. This was reiterated in the report.^13^ Efforts relating to HIV screening and testing, as well as condom distribution amongst older men, need to be scaled up to ensure they do not continue spreading the infection to their older and younger female sexual partners. Another intervention strategy could include a mass medical male circumcision campaign, aimed specifically at older men.

The majority of this study cohort had been on ART for ≤ 10 years. It was evident from the file reviews that changing treatment guidelines over the years has had an influence on ART initiation in this population, as they were more likely to be initiated in line with their CD4 cell count values at a later stage of infection. Dates of HIV diagnoses were unavailable for many participants, which made it impossible to report on the timeline from receiving a diagnosis of HIV and initiating treatment. In the era of UTT, ART should be initiated as soon as possible for all those who qualify for it, especially in OPLWH. This could potentially negate a poor immune reconstitution effort and improve the overall clinical status for OPLWH. Moreover, this could limit the spread of the disease to their sexual partners. This may also empower them to learn more about the disease and educate their families about it.

It may be challenging to comment on the current ART regimen of this cohort, as it was unclear if and when regimens were changed from a stavudine-based regimen for those taking treatment for longer, as per evolving guidelines. It was clear, however, that an NNRTI-based regimen was the most common treatment regimen prescribed for these participants in line with guidelines used at the time. The tenofovir component was not included in 16.2% of participants’ ART and 6.8% of the cohort had renal dysfunction. Only 3.7% of the cohort was classified with CKD. One explanation for the low prevalence of CKD may be that renal function improved when tenofovir was substituted with an agent that is known to be renal protective, and these participants were subsequently not classified as having CKD, but this does not explain the higher percentage of renal dysfunction in this cohort. Another explanation may be that primary care clinicians simply did not diagnose or document CKD in this cohort. The prevalence of CKD was one in five PLWH ≥ 60 years old in a study conducted in 12 French hospitals and was independently associated with an increased 5-year mortality.^22^ These findings may warrant more rigorous screening and documentation of kidney function in our setting, especially in light of the fact that CKD is a known risk factor for adverse cardiovascular outcomes.^23^

Knowing the nutritional status of a patient might affect 21% – 82% of treatment decisions,^24^ with poor nutritional status strongly associated with mortality.^25^ The presence of anaemia and poor nutritional status are interconnected, and in this cohort the presence of anaemia was associated with the risk of dying. Unfortunately, height and thus body mass index (BMI) and/or mid-upper arm circumference (MUAC) measurements were not routinely measured in the care of this study’s participants. Haemoglobin levels were also not readily available for this cohort. The weight (median and IQR) nevertheless correlated well with that of participants in another South African study.^9^ Nutritional status is one of the components that needs to be assessed in a comprehensive geriatric assessment (CGA). This tool includes 11 components that address biomedical, social and economic concerns for HIV care providers relating to OPLWH.^24^ Recent hospitalisation is a known risk factor for dying in OPLWH, and this was echoed in this study. A meta-analysis showed that those who underwent a CGA whilst hospitalised were more likely to be alive after 12 months than those who did not.^25^ An explanation for this may be that the teams who performed CGA were more experienced and specialised than the teams who typically worked on the wards. Long-term follow-up also appeared to be more comprehensive in the participants who underwent CGA in the hospital setting. This is a novel tool in HIV care; it has been used successfully in other disciplines and may prove to be a crucial tool in the HIV care sphere pertaining to the ageing population in years to come.^26^ Hence, there are ample reasons for incorporating anthropometric measures, including those related to nutritional status and anaemia, into the clinical guide for the care of OPLWH. However, clinicians in better resourced settings are struggling to adhere to CGA recommendations^24^ and its rollout to resource-constrained settings may come with challenges, such as lack of experience with the tool, time and resource limitations and insufficient evidence for its effectiveness in African settings.

Even in this reasonably small cohort, the prevalence of HPT and DM were high compared with other studies.^9,17^ In these studies, the prevalence of HPT ranged from 21.5% – 33.3%, the latter percentage being for participants aged ≥ 70 years. In our study population, there were 55.5% of OPLWH on treatment for HPT. The differences may be due to the other studies actively measuring participants’ blood pressure at ART initiation, whereas the current study relied on clinical records and could not account for what might have come first: HIV or HPT. The same could be said about DM, where the prevalence ranged from 2.2% – 6.3% in the other cohorts, compared to 7.9% in the current cohort. Again, conclusive interpretation of these results is elusive because the current study inception was not at ART initiation. It is well established that ART and ageing both accelerate cardiovascular disease risk in older adults.^8^ Added to the cardiovascular disease burden of HPT, DM and CKD, it stands to reason that older adults require tailor-made interventions to address their cardiovascular health. The administration of a novel polypill (including a statin, aspirin and anti-HPT medications) is a potential option to reduce cardiovascular and cancer morbidity and mortality in OPLWH.^8^

The VL suppression in this cohort was lower than previously reported in SA.^9,10,16^ Moreover, VL suppression was attained in 86% – 89.5% of older adults in other settings after 12–60 months of treatment. It was perturbing that only 81.9% of this cohort had a suppressed VL, especially as a larger proportion of them were on second-line treatment than found in another South African cohort (5.2% versus 0.88)^16^; and female participants, who are historically more adherent to treatment, had lower VL suppression rates. Only 5.2% of the cohort were on second-line ART. The rest were still on a NNRTI-based regimen. Moreover, over 85% of those with an unsuppressed VL were still on an NNRTI-based regimen. HIV treatment and monitoring guidelines in SA have changed since this study was conducted. The new guidelines define a suppressed VL as < 50 copies/ml.^27^ If these criteria were used to assess VL suppression, only 59% of this cohort would be suppressed. Although high-range low-level viraemia (VL 400 copies/mL – 999 copies/mL) in this cohort was low (8.4%), a previous study performed in SA showed a five-times increased risk of virological failure in participants who had VL readings in this range.^28^ It is imperative to monitor these patients closely and act appropriately if and when true virological failure (VL ≥ 1000 copies/mL) and attenuated CD4 cells count responses develop. Appropriate action, that is changing to a second-line ART regimen, seemed to be lacking in this cohort. There was, likely, treatment failure and the development of resistance to ART in this cohort. This may hamper the 90–90–90 UNAIDS target of achieving a 90% VL suppression rate in all age groups globally by 2030. South Africa currently stands at 87% VL suppression in 54% of all PLWH.^29^ More attention should be given to OPLWH in order to attain the UNAIDS goals and ensure their overall well-being and prevent the spread of drug-resistant HIV.

Malaza et al.^30^ found that the median CD4 cell count in OPLWH was 367 cells/mm^3^ after a median duration of 2.3 years on ART. Similarly, Fatti et al.^16^ found that OPLWH had a median CD4 cell count of 377 cells/mm^3^ after 3 years on ART. The median CD4 cell count increased by 256.5 cells/mm^3^ from the baseline in this cohort. Fatti et al.^16^ also found that the median CD4 cell count increased from about 100 cells/mm^3^ after 6 months to over 300 cells/mm^3^ after 48 months, since ART initiation. It is well-established that CD4 cell recovery is attenuated in OPLWH compared to younger PLWH.^16,30^ In this study, it was evident that higher recent CD4 cell counts were more likely in those who had been taking ART for > 5 years (OR = 3.15, 95% CI 1.34–7.40, *p* = 0.009) and those on the usual first-line ART (OR = 2.78, 95% CI 1.04–7.42, *p* = 0.041). The former may have been an obvious finding because of improved immune reconstitution, the sooner a patient is initiated on ART,^8^ but further work may be needed to evaluate the latter.

## Strengths and limitations

Most previous studies in the field of OPLWH have defined them to be aged ≥ 50 years. However, seeing as assumptions of life expectancy without HIV and total fertility rates were estimated to be 61.5 years for males and 67.7 years for females in 2019,^13^ it was imperative to assess older age groups and exclude those still eligible to work for future planning of care interventions for these individuals. This is one of the first local studies to use this novel approach. Also, a paucity of data was available on the subject in the NWP according to the researcher’s knowledge, and this is the first study to begin to address this gap. Most previous studies published about OPLWH have compared their response since ART initiation to younger cohorts. The current study did not address this. The sample was chosen conveniently and thus could have introduced selection bias. Also, the retrospective design has disadvantages. Previous prospective studies on the subject could compare baseline characteristics and outcomes in terms of ART initiation and follow-up. This approach was not possible in the current study due to time constraints in terms of data collection, and there were many missing data (e.g. files and blood results) which could have influenced study findings. The sample size was small and this could perhaps be seen as a pilot study for future studies in OPLWH in the NWP. Generalisability was affected by limited patient numbers and the exclusion of a more rural cohort. Unmeasured covariates could have resulted in hidden bias.

## Conclusion

To our knowledge, this was the first study to assess characteristics and outcomes in older PLWH in the NWP. Older men and women living with HIV need to be approached using different strategies, tailored to their specific needs. There is an urgent need for South African clinicians to consider the implementation of standardised CGA in all OPLWH, as this has the potential to improve outcomes of older patients after hospital admission. Chronic kidney disease needs to be actively excluded in OPLWH as it has major clinical implications, especially in the context of a high burden of other co-morbidities and high cardiovascular morbidity and mortality. Considering the study limitations, further prospective studies with larger samples are necessary in the NWP to confirm or refute the findings of low VL suppression rates in OPLWH.

## Appendix 1: Study definitions

- **Previous defaulter:** Past single or serial failure by the patient to collect his or her treatment as documented in the clinical record or on TIER.Net.
- **Ward-based outreach team (WBOT):** A clinic-based team that collected treatment on the patient’s behalf at any time during the study period. A patient could thus be categorised in one, two or three categories in terms of treatment collection during the study period.
- **Transfer/move out as per clinical record or TIER.Net:** A distinction was made about whether or not the new treatment collection site was still the primary care facility or at a higher level of care.
- **Co-morbidity as documented on ART stationery:** If the co-morbidity was unstated but treatment was prescribed during the period under review at any time, the patient was considered as having the related co-morbidity.
- **Recent hospitalisation:** A discharge summary spanning the previous 12 months recorded in the patient file or specified in clinical notes.
- **Suppressed viral load:** A reading of < 400 copies/mL.
- **Adequate immunological response:** A CD4 cell count of ≥ 350 cells/mm^3^ within 1 year of the data census being documented in the clinical record.
- **Loss to follow-up:** The patient’s treatment was not collected within 3 months (90 days) of the latest (last) study visit at the study census and the patient could not be traced either telephonically or at his or her place of residence by community health workers during data collection period.
- **Died:** The patient’s date of death was documented in the clinical records or on TIER.Net.
